# Supplementary material for: Common variation in a long non-coding RNA gene modulates variation of circulating TGF-β2 levels in metastatic colorectal cancer patients (Alliance)
Source: BMC Genomics. 2024 May 14;25:473. doi: 10.1186/s12864-024-10354-7 (PMC11092225; doi:10.1186/s12864-024-10354-7)
Supplement: Supplementary file 2 — Supplementary Material 2 [file 12864_2024_10354_MOESM2_ESM.html]

|  | TGFb1 | TGF-b2 | VEGF-D | OPN | PDGF-AA | IL-6 | VEGF-R1 | TSP2 | VEGF-R3 | Ang-2 | ICAM-1 | TIMP-1 | VCAM-1 | HGF | PDGF-BB | VEGF-A | PlGF | VEGF-R2 | SDF-1 | BMP-9 | CD73 | HER-3 | TGFb-R3 |
| --- | --- | --- | --- | --- | --- | --- | --- | --- | --- | --- | --- | --- | --- | --- | --- | --- | --- | --- | --- | --- | --- | --- | --- |
| 1 | rs2684781 4.7e-06 | rs11118119 1.4e-14 | rs3923808 7.5e-06 | rs1053000 3.0e-06 | rs2684781 2.3e-06 | rs8106493 8.0e-06 | rs2108258 1.9e-06 | rs6945041 8.7e-07 | rs10935473 6.4e-39 | rs4242626 1.4e-06 | rs6945041 2.4e-06 | rs17111972 2.8e-06 | rs7781339 2.8e-07 | rs2684879 9.6e-06 | rs2192925 1.7e-06 | rs7767396 2.6e-12 | rs11117450 2.9e-06 | rs881944 6.7e-08 | rs17219447 2.7e-06 | rs2588599 6.6e-06 | rs3812138 2.3e-15 | rs2923419 6.9e-06 | rs4645549 1.6e-06 |
| 2 | rs11158890 4.9e-06 | rs12034138 1.8e-14 | rs684584 7.9e-06 | rs2279216 5.7e-06 | rs12215704 4.1e-06 | rs1654654 9.7e-06 | rs13116766 3.1e-06 | rs10521067 6.3e-06 | rs4857414 1.7e-32 | rs7867504 1.5e-06 | rs408005 4.5e-06 | rs6945041 3.2e-06 | rs6945041 3.6e-07 | rs6597 1.1e-05 | rs10743555 2.8e-06 | rs9472159 3.8e-11 | rs3747846 7.0e-06 | rs6816309 1.8e-07 | rs3757030 4.2e-06 | rs2588604 8.3e-06 | rs6922 1.5e-14 | rs2297738 8.5e-06 | rs10766560 2.0e-06 |
| 3 | rs9405157 6.4e-06 | rs6665024 3.3e-12 | rs7201920 9.1e-06 | rs1217793 1.1e-05 | rs9405157 4.8e-06 | rs357405 1.2e-05 | rs12907953 4.7e-06 | rs920809 1.0e-05 | rs3804622 5.7e-23 | rs10785733 3.3e-06 | rs150822 5.8e-06 | rs6106161 1.2e-05 | rs6972755 5.6e-07 | rs10853347 1.3e-05 | rs12595754 2.8e-06 | rs9369434 1.2e-09 | rs10196764 1.2e-05 | rs687289 2.4e-07 | rs12593336 5.2e-06 | rs1436652 1.5e-05 | rs2842608 2.8e-14 | rs17875558 1.4e-05 | rs7844947 3.2e-06 |
| 4 | rs2904493 8.9e-06 | rs725033 1.6e-10 | rs713532 9.5e-06 | rs2306311 1.1e-05 | rs7865979 1.3e-05 | rs10444498 1.4e-05 | rs521184 5.2e-06 | rs4742901 1.1e-05 | rs6797163 3.3e-20 | rs4877829 7.2e-06 | rs9300791 8.0e-06 | rs2760311 1.7e-05 | rs12229292 1.7e-06 | rs1844346 1.4e-05 | rs9980087 3.2e-06 | rs9472158 2.3e-07 | rs2501657 1.3e-05 | rs10187088 2.7e-07 | rs6911180 5.9e-06 | rs676715 1.7e-05 | rs9444361 1.1e-13 | rs17875556 1.5e-05 | rs815804 3.3e-06 |
| 5 | rs12215704 1.5e-05 | rs11118103 1.4e-08 | rs7661528 9.7e-06 | rs8058889 1.2e-05 | rs1125467 1.3e-05 | rs929878 1.6e-05 | rs1844346 8.9e-06 | rs11154583 1.2e-05 | rs844159 1.1e-19 | rs4562384 8.5e-06 | rs375154 9.4e-06 | rs910514 1.8e-05 | rs4981021 5.7e-06 | rs1769207 1.5e-05 | rs741321 3.3e-06 | rs729391 9.6e-07 | rs11582243 1.6e-05 | rs12331869 2.8e-07 | rs11837160 6.2e-06 | rs479359 1.7e-05 | rs6931295 1.3e-13 | rs567801 1.5e-05 | rs1370446 5.5e-06 |
| 6 | rs6016883 1.8e-05 | rs6604616 2.2e-08 | rs1932033 9.8e-06 | rs75488 1.7e-05 | rs171010 1.4e-05 | rs212097 2.1e-05 | rs13132077 1.3e-05 | rs2760311 1.2e-05 | rs865474 3.3e-19 | rs4790839 1.1e-05 | rs2896135 1.1e-05 | rs9285466 1.9e-05 | rs6575802 6.3e-06 | rs1510324 1.9e-05 | rs7917062 5.0e-06 | rs7764227 1.8e-06 | rs4916185 1.6e-05 | rs13126206 4.2e-07 | rs7624183 7.5e-06 | rs4689547 1.9e-05 | rs4593336 2.7e-12 | rs4877498 1.5e-05 | rs4873544 5.8e-06 |
| 7 | rs6031255 1.9e-05 | rs6501554 9.9e-07 | rs10838235 1.4e-05 | rs17501032 1.8e-05 | rs6031255 1.8e-05 | rs2569006 2.7e-05 | rs714026 1.4e-05 | rs131833 1.2e-05 | rs4558798 2.6e-15 | rs12480063 1.2e-05 | rs7025066 1.2e-05 | rs17501032 2.0e-05 | rs10510509 1.1e-05 | rs2108258 2.1e-05 | rs2777531 5.5e-06 | rs1953105 2.1e-06 | rs4852213 1.7e-05 | rs657152 1.1e-06 | rs6519141 7.8e-06 | rs417664 2.0e-05 | rs7752502 3.9e-12 | rs17875557 1.5e-05 | rs7827306 7.5e-06 |
| 8 | rs28384 2.2e-05 | rs10088057 1.6e-06 | rs113080 1.7e-05 | rs2311719 1.9e-05 | rs6893444 2.0e-05 | rs335598 2.8e-05 | rs9938218 1.6e-05 | rs1364779 1.5e-05 | rs1675526 3.6e-15 | rs10485497 1.2e-05 | rs7644823 1.2e-05 | rs100537 2.2e-05 | rs100537 1.2e-05 | rs8018565 2.4e-05 | rs945254 6.0e-06 | rs4961557 2.7e-06 | rs1554220 1.9e-05 | rs507666 2.0e-06 | rs1437221 8.2e-06 | rs6833202 2.3e-05 | rs12212560 8.3e-12 | rs7303424 1.8e-05 | rs843899 7.8e-06 |
| 9 | rs3912148 2.3e-05 | rs6703224 2.7e-06 | rs2968933 1.9e-05 | rs11847083 2.1e-05 | rs2023748 2.1e-05 | rs2026882 2.9e-05 | rs10164725 1.7e-05 | rs11221124 2.2e-05 | rs507666 9.1e-12 | rs4014085 1.4e-05 | rs988956 1.3e-05 | rs9290557 2.8e-05 | rs3118941 1.9e-05 | rs8066066 2.4e-05 | rs10806415 6.7e-06 | rs4506764 5.3e-06 | rs12518914 2.0e-05 | rs1515931 2.9e-06 | rs7559552 1.2e-05 | rs2192599 2.6e-05 | rs9362210 1.4e-11 | rs7241000 2.1e-05 | rs4471026 7.9e-06 |
| 10 | rs6893444 2.4e-05 | rs1473527 4.2e-06 | rs12448859 2.0e-05 | rs12293872 2.2e-05 | rs41737 2.1e-05 | rs13114604 2.9e-05 | rs1338203 1.9e-05 | rs4770399 2.3e-05 | rs579459 9.2e-12 | rs1963996 1.6e-05 | rs2300496 1.4e-05 | rs6431749 3.2e-05 | rs17484467 2.1e-05 | rs6062129 2.7e-05 | rs10827214 1.2e-05 | rs2044119 6.0e-06 | rs17040199 2.3e-05 | rs11055416 4.1e-06 | rs13201804 1.5e-05 | rs12699981 3.3e-05 | rs494688 1.9e-09 | rs1548774 2.3e-05 | rs7827446 1.0e-05 |
| 11 | rs11654128 2.4e-05 | rs1891467 4.3e-06 | rs178525 2.2e-05 | rs1217809 2.5e-05 | rs41738 2.1e-05 | rs632023 3.1e-05 | rs10028425 2.0e-05 | rs4758259 2.7e-05 | rs7630058 2.3e-11 | rs1418479 1.8e-05 | rs6972755 1.5e-05 | rs320201 4.0e-05 | rs1128977 2.4e-05 | rs10028425 3.1e-05 | rs1360456 1.2e-05 | rs1591138 6.2e-06 | rs2685211 2.7e-05 | rs2219469 4.4e-06 | rs13334022 2.3e-05 | rs10104374 3.8e-05 | rs1414201 2.3e-09 | rs831612 2.6e-05 | rs2196427 1.5e-05 |
| 12 | rs11845762 2.4e-05 | rs6657275 5.2e-06 | rs1007634 2.5e-05 | rs17220520 2.8e-05 | rs6566 2.1e-05 | rs4695845 3.1e-05 | rs9312664 2.1e-05 | rs12925126 2.7e-05 | rs7628381 3.1e-11 | rs2275420 2.0e-05 | rs1850126 1.5e-05 | rs10409533 4.1e-05 | rs1440887 2.4e-05 | rs1487336 3.1e-05 | rs6717927 1.2e-05 | rs331595 6.8e-06 | rs12736367 2.7e-05 | rs12464563 4.8e-06 | rs8015527 2.3e-05 | rs4871364 3.9e-05 | rs10455449 6.0e-09 | rs11876129 3.1e-05 | rs6550509 1.5e-05 |
| 13 | rs9558485 2.5e-05 | rs10104370 5.8e-06 | rs4771482 2.6e-05 | rs702524 3.0e-05 | rs2192925 2.1e-05 | rs681653 3.1e-05 | rs966867 2.1e-05 | rs4562384 3.0e-05 | rs3755569 4.5e-11 | rs2486118 2.2e-05 | rs4883879 1.8e-05 | rs480211 5.4e-05 | rs17111972 2.4e-05 | rs16915839 3.2e-05 | rs2192926 1.3e-05 | rs7238524 8.7e-06 | rs2970533 3.1e-05 | rs2071559 5.4e-06 | rs2008555 2.5e-05 | rs2111168 4.0e-05 | rs12200330 6.2e-09 | rs7687690 3.5e-05 | rs4500070 2.3e-05 |
| 14 | rs623489 2.8e-05 | rs9355442 7.5e-06 | rs1909143 2.7e-05 | rs17220853 3.2e-05 | rs41739 2.1e-05 | rs2194079 3.7e-05 | rs12376441 2.2e-05 | rs7025066 3.1e-05 | rs7103 1.3e-10 | rs2632487 2.4e-05 | rs12758064 1.8e-05 | rs1479831 5.5e-05 | rs375154 2.5e-05 | rs11107485 3.3e-05 | rs2023748 1.8e-05 | rs10962988 9.3e-06 | rs2305599 3.2e-05 | rs8913 7.2e-06 | rs12446055 2.9e-05 | rs10486157 4.2e-05 | rs12199516 6.2e-09 | rs868142 3.6e-05 | rs17488695 2.4e-05 |
| 15 | rs2023748 3.1e-05 | rs1491457 8.0e-06 | rs10063429 2.7e-05 | rs910514 3.3e-05 | rs828644 2.4e-05 | rs807293 4.0e-05 | rs13394510 2.2e-05 | rs9285466 3.2e-05 | rs9825798 2.0e-10 | rs2272631 2.5e-05 | rs11058222 2.2e-05 | rs9845152 6.2e-05 | rs471071 2.5e-05 | rs13004808 3.5e-05 | rs41737 1.8e-05 | rs2593382 1.2e-05 | rs1179911 3.2e-05 | rs12918979 8.2e-06 | rs10493166 2.9e-05 | rs6956624 4.2e-05 | rs2121594 7.7e-09 | rs1550202 3.7e-05 | rs4873189 2.5e-05 |
| 16 | rs41737 3.1e-05 | rs7459665 8.1e-06 | rs17455138 2.8e-05 | rs9951539 3.5e-05 | rs2421932 2.4e-05 | rs12229047 4.0e-05 | rs7822830 2.2e-05 | rs1425865 3.2e-05 | rs1531377 4.1e-10 | rs10441725 2.6e-05 | rs11017735 2.5e-05 | rs2124710 6.2e-05 | rs9876711 2.9e-05 | rs3738919 3.7e-05 | rs41738 1.8e-05 | rs10121841 1.4e-05 | rs11757455 3.3e-05 | rs2233537 9.7e-06 | rs6001258 3.0e-05 | rs2741675 4.3e-05 | rs6913634 1.2e-08 | rs1414738 3.9e-05 | rs1549688 2.7e-05 |
| 17 | rs41738 3.1e-05 | rs6845803 8.1e-06 | rs10078467 2.8e-05 | rs6431749 4.4e-05 | rs12363522 2.5e-05 | rs2408868 4.0e-05 | rs274555 2.3e-05 | rs12432710 4.0e-05 | rs1000003 6.9e-10 | rs536937 2.6e-05 | rs4551296 3.9e-05 | rs7602635 6.4e-05 | rs869611 2.9e-05 | rs13149803 4.0e-05 | rs6566 1.8e-05 | rs2837907 1.5e-05 | rs4297406 3.5e-05 | rs850813 1.6e-05 | rs8141420 3.1e-05 | rs45430 4.8e-05 | rs1447157 1.3e-08 | rs574865 3.9e-05 | rs2035985 2.8e-05 |
| 18 | rs6566 3.1e-05 | rs1886090 1.0e-05 | rs1909147 2.9e-05 | rs4798740 4.7e-05 | rs3930083 2.7e-05 | rs3795026 4.3e-05 | rs2060774 2.4e-05 | rs11017735 4.3e-05 | rs7653204 9.2e-10 | rs10515839 2.7e-05 | rs761878 4.1e-05 | rs1774037 6.6e-05 | rs751448 3.0e-05 | rs2684880 4.2e-05 | rs41739 1.8e-05 | rs1500321 1.5e-05 | rs951204 3.5e-05 | rs3849934 1.7e-05 | rs3735361 3.2e-05 | rs11068917 5.5e-05 | rs9342047 1.6e-08 | rs2029356 4.0e-05 | rs9298444 3.2e-05 |
| 19 | rs41739 3.1e-05 | rs171010 1.0e-05 | rs10517087 2.9e-05 | rs2903582 5.2e-05 | rs11158890 2.7e-05 | rs2546082 4.6e-05 | rs7202483 2.6e-05 | rs6972755 4.4e-05 | rs4857412 1.1e-09 | rs4746080 2.7e-05 | rs4264108 4.6e-05 | rs761878 6.7e-05 | rs4964830 3.0e-05 | rs1338203 4.6e-05 | rs2188994 2.8e-05 | rs654521 1.9e-05 | rs2340022 3.6e-05 | rs4702147 1.9e-05 | rs10462511 3.4e-05 | rs4528301 5.6e-05 | rs6900270 1.9e-08 | rs3120803 4.0e-05 | rs10093574 3.4e-05 |
| 20 | rs6031256 3.3e-05 | rs17457575 1.2e-05 | rs6571631 3.2e-05 | rs6972755 5.7e-05 | rs2544037 2.8e-05 | rs349353 5.0e-05 | rs4553153 2.6e-05 | rs6590292 4.8e-05 | rs1729963 1.3e-08 | rs1332538 3.0e-05 | rs849346 4.7e-05 | rs17059066 6.7e-05 | rs3812805 3.0e-05 | rs2452928 4.7e-05 | rs733610 2.8e-05 | rs9539140 2.0e-05 | rs460248 3.7e-05 | rs7149767 2.0e-05 | rs16948906 3.4e-05 | rs33385 6.5e-05 | rs9362215 2.1e-08 | rs10272254 4.0e-05 | rs4322600 3.7e-05 |
| 21 | rs6016884 3.4e-05 | rs17350701 1.2e-05 | rs1995720 3.4e-05 | rs1031348 5.8e-05 | rs12491294 3.0e-05 | rs854864 5.3e-05 | rs10049987 2.7e-05 | rs2975398 5.0e-05 | rs1675505 9.0e-08 | rs10046030 3.0e-05 | rs7779963 4.7e-05 | rs805757 6.9e-05 | rs2183644 3.1e-05 | rs6577604 5.0e-05 | rs41736 2.9e-05 | rs10815828 2.3e-05 | rs16951897 3.9e-05 | rs9655857 2.1e-05 | rs1720866 3.6e-05 | rs4766899 6.6e-05 | rs9450383 2.4e-08 | rs11639206 4.1e-05 | rs11745459 4.1e-05 |
| 22 | rs12881813 3.8e-05 | rs6683598 1.3e-05 | rs2940288 3.5e-05 | rs17162231 6.0e-05 | rs11654128 3.3e-05 | rs4247362 5.3e-05 | rs848039 2.8e-05 | rs150822 5.8e-05 | rs11552978 1.3e-07 | rs984659 3.2e-05 | rs35974282 4.8e-05 | rs12293872 7.1e-05 | rs2271421 3.2e-05 | rs17235684 5.2e-05 | rs12215704 3.0e-05 | rs10511491 2.4e-05 | rs706341 4.2e-05 | rs11055424 2.1e-05 | rs10493168 3.8e-05 | rs955013 6.6e-05 | rs6942065 3.2e-08 | rs1912124 4.3e-05 | rs10864471 4.6e-05 |
| 23 | rs4558315 3.9e-05 | rs2880363 1.6e-05 | rs1002252 3.8e-05 | rs7025066 6.1e-05 | rs6031256 4.0e-05 | rs1791657 5.7e-05 | rs12043594 2.9e-05 | rs408005 5.8e-05 | rs4857097 1.8e-07 | rs2894147 3.3e-05 | rs2479691 4.9e-05 | rs817095 7.2e-05 | rs1852491 3.2e-05 | rs8135478 5.2e-05 | rs2826958 3.2e-05 | rs9490542 2.4e-05 | rs17436982 4.5e-05 | rs7821974 2.4e-05 | rs11856427 3.8e-05 | rs7864179 6.9e-05 | rs9353319 3.6e-08 | rs10183727 4.5e-05 | rs10058233 4.6e-05 |
| 24 | rs7865979 4.0e-05 | rs2328452 2.4e-05 | rs9422868 3.8e-05 | rs10510535 6.1e-05 | rs1192041 4.0e-05 | rs10812662 5.7e-05 | rs10846382 2.9e-05 | rs16928804 6.3e-05 | rs6778616 2.1e-07 | rs9543601 3.5e-05 | rs1573993 5.0e-05 | rs1536982 7.4e-05 | rs892542 3.4e-05 | rs10409533 5.2e-05 | rs2826973 3.4e-05 | rs13155924 2.6e-05 | rs12477177 4.6e-05 | rs7599354 2.4e-05 | rs4345300 3.9e-05 | rs2328406 7.4e-05 | rs9859162 1.0e-06 | rs2486011 4.7e-05 | rs9883654 4.8e-05 |
| 25 | rs1125467 4.0e-05 | rs4712586 2.5e-05 | rs529802 4.0e-05 | rs1289392 6.3e-05 | rs623489 4.1e-05 | rs951772 5.8e-05 | rs10992979 3.2e-05 | rs6431749 6.4e-05 | rs1675511 2.3e-07 | rs7316 3.6e-05 | rs224630 5.0e-05 | rs768566 8.2e-05 | rs12407970 3.4e-05 | rs2452922 5.6e-05 | rs11165315 3.5e-05 | rs7553537 2.7e-05 | rs911112 4.6e-05 | rs7644659 2.4e-05 | rs17107212 4.2e-05 | rs10007790 7.4e-05 | rs9450325 1.6e-06 | rs6827352 4.8e-05 | rs803054 5.5e-05 |
| 26 | rs6030450 4.2e-05 | rs10409533 2.5e-05 | rs9422871 4.1e-05 | rs6570675 6.7e-05 | rs903336 4.3e-05 | rs1732305 5.8e-05 | rs13149803 3.2e-05 | rs1560034 6.7e-05 | rs1350789 3.1e-07 | rs6885337 3.9e-05 | rs131833 5.0e-05 | rs13100398 8.8e-05 | rs11980671 3.4e-05 | rs2635304 6.3e-05 | rs1625694 3.8e-05 | rs2779773 2.8e-05 | rs7088954 4.7e-05 | rs12502008 2.6e-05 | rs11818345 4.6e-05 | rs7749330 7.5e-05 | rs2273265 4.4e-06 | rs9514742 4.9e-05 | rs11014799 5.6e-05 |
| 27 | rs4812640 4.3e-05 | rs1479831 2.6e-05 | rs4148650 4.5e-05 | rs9316279 7.0e-05 | rs7917062 4.3e-05 | rs4792606 5.8e-05 | rs6577599 3.3e-05 | rs11058254 7.3e-05 | rs657152 4.2e-07 | rs4560590 3.9e-05 | rs10139382 5.1e-05 | rs7647575 8.8e-05 | rs4895290 3.5e-05 | rs748731 6.5e-05 | rs17179453 4.1e-05 | rs4712108 2.9e-05 | rs7182543 4.7e-05 | rs4956410 2.6e-05 | rs7717415 5.0e-05 | rs6928245 7.7e-05 | rs3746674 4.7e-06 | rs12591927 4.9e-05 | rs7706539 5.7e-05 |
| 28 | rs10163323 4.3e-05 | rs4846483 3.1e-05 | rs646372 4.5e-05 | rs10816284 7.1e-05 | rs741321 4.3e-05 | rs7481121 5.9e-05 | rs13083429 3.3e-05 | rs4896668 7.4e-05 | rs687289 5.3e-07 | rs2081957 3.9e-05 | rs12407970 5.1e-05 | rs2145572 8.9e-05 | rs944467 3.5e-05 | rs17288525 6.5e-05 | rs818442 4.1e-05 | rs9397510 2.9e-05 | rs3802902 5.1e-05 | rs138087 2.7e-05 | rs4704197 5.1e-05 | rs431563 7.7e-05 | rs4790839 5.4e-06 | rs10885912 5.3e-05 | rs6891720 5.7e-05 |
| 29 | rs1022818 4.4e-05 | rs2537978 3.1e-05 | rs8067791 4.5e-05 | rs131833 7.6e-05 | rs10073138 4.4e-05 | rs1536982 5.9e-05 | rs11960862 3.4e-05 | rs2241571 7.7e-05 | rs558240 7.9e-07 | rs4877842 3.9e-05 | rs6575129 5.4e-05 | rs9672615 9.2e-05 | rs6082932 3.8e-05 | rs738483 6.7e-05 | rs9366347 4.2e-05 | rs1487452 2.9e-05 | rs10884462 5.4e-05 | rs579459 2.7e-05 | rs2268199 5.7e-05 | rs4689050 7.8e-05 | rs220402 7.4e-06 | rs2797349 5.3e-05 | rs2420998 5.7e-05 |
| 30 | rs17700949 4.7e-05 | rs2827079 3.2e-05 | rs3759236 4.7e-05 | rs7994542 7.7e-05 | rs9392354 4.6e-05 | rs537172 5.9e-05 | rs7533806 3.5e-05 | rs310011 7.8e-05 | rs7431448 9.2e-07 | rs1914159 4.0e-05 | rs7213894 5.6e-05 | rs17197715 9.6e-05 | rs17501032 3.8e-05 | rs2595389 6.7e-05 | rs853926 4.3e-05 | rs2012074 3.1e-05 | rs6881927 5.7e-05 | rs4356965 3.0e-05 | rs1600074 5.7e-05 | rs2629812 8.2e-05 | rs9450286 9.1e-06 | rs2107901 5.6e-05 | rs10409452 5.9e-05 |
| 31 | rs12953270 4.8e-05 | rs12044712 3.2e-05 | rs1567192 4.7e-05 | rs9877584 8.1e-05 | rs6595591 4.7e-05 | rs1563400 5.9e-05 | rs6577604 3.7e-05 | rs4833025 7.9e-05 | rs1569234 1.3e-06 | rs325234 4.1e-05 | rs741554 5.9e-05 | rs10852106 9.6e-05 | rs9852553 3.8e-05 | rs12043594 6.8e-05 | rs6496898 4.3e-05 | rs9914435 3.1e-05 | rs4430760 5.9e-05 | rs10517342 3.0e-05 | rs1422698 5.7e-05 | rs2616972 8.3e-05 | rs10270197 9.4e-06 | rs4571555 5.6e-05 | rs1807821 6.0e-05 |
| 32 | rs6566386 4.9e-05 | rs7303188 3.3e-05 | rs1263360 4.9e-05 | rs886374 9.0e-05 | rs2237796 4.8e-05 | rs9668031 6.0e-05 | rs1742500 4.1e-05 | rs6776145 8.2e-05 | rs11489075 2.9e-06 | rs6785267 4.9e-05 | rs1379561 6.1e-05 | rs3742245 9.7e-05 | rs9132 4.0e-05 | rs1604643 6.9e-05 | rs1882347 4.4e-05 | rs854555 3.3e-05 | rs3862550 6.3e-05 | rs2241998 3.0e-05 | rs2216638 5.7e-05 | rs354009 8.6e-05 | rs10944128 1.0e-05 | rs1542601 5.7e-05 | rs10762307 6.3e-05 |
| 33 | rs7304605 5.2e-05 | rs1113253 3.7e-05 | rs2860199 5.0e-05 | rs285823 9.1e-05 | rs1381097 4.9e-05 | rs10861954 6.0e-05 | rs13042362 4.2e-05 | rs1379561 8.4e-05 | rs1729964 3.1e-06 | rs17164103 5.1e-05 | rs17476273 6.1e-05 | rs11265346 9.8e-05 | rs10861096 4.0e-05 | rs966867 7.0e-05 | rs6992898 4.9e-05 | rs9372752 3.3e-05 | rs13220356 6.5e-05 | rs331723 3.3e-05 | rs1477937 5.7e-05 | rs354013 8.6e-05 | rs3807294 1.1e-05 | rs4144709 6.3e-05 | rs9868777 6.5e-05 |
| 34 | rs9392354 5.2e-05 | rs570098 3.9e-05 | rs3803704 5.2e-05 | rs6561362 9.6e-05 | rs41736 5.0e-05 | rs11007129 6.1e-05 | rs17288525 4.3e-05 | rs12831504 8.4e-05 | rs7755288 5.2e-06 | rs2505176 5.5e-05 | rs899541 6.2e-05 | rs1456610 1.0e-04 | rs10861097 4.0e-05 | rs11735191 7.2e-05 | rs2027605 4.9e-05 | rs4984422 3.5e-05 | rs10122844 6.7e-05 | rs2147753 3.3e-05 | rs10462354 6.1e-05 | rs11034323 8.8e-05 | rs10263650 1.3e-05 | rs17027998 6.3e-05 | rs4746896 6.6e-05 |
| 35 | rs12626167 5.3e-05 | rs9653629 4.1e-05 | rs754469 5.2e-05 | rs3802780 9.7e-05 | rs2237790 5.2e-05 | rs349335 6.1e-05 | rs4654402 4.3e-05 | rs11641366 8.6e-05 | rs9853063 5.7e-06 | rs1934158 6.0e-05 | rs10479243 6.2e-05 | rs7162568 1.0e-04 | rs1288518 4.0e-05 | rs9672615 7.4e-05 | rs2346732 5.0e-05 | rs10822288 3.5e-05 | rs11212939 6.8e-05 | rs342511 3.4e-05 | rs1552621 6.3e-05 | rs11753925 8.8e-05 | rs7824350 1.7e-05 | rs10772890 6.4e-05 | rs1572340 6.8e-05 |
| 36 | rs2033538 5.4e-05 | rs6112574 4.1e-05 | rs903951 5.4e-05 | rs805757 9.7e-05 | rs9391943 5.5e-05 | rs7788668 6.6e-05 | rs2908201 4.8e-05 | rs11221112 8.9e-05 | rs1062196 6.2e-06 | rs10868145 6.1e-05 | rs6479222 6.3e-05 | rs131833 1.0e-04 | rs10217136 4.2e-05 | rs982443 8.0e-05 | rs11169752 5.1e-05 | rs4262389 3.9e-05 | rs17099789 7.0e-05 | rs3949927 3.4e-05 | rs17276794 6.6e-05 | rs17105816 8.9e-05 | rs9472892 1.7e-05 | rs235626 6.9e-05 | rs2812541 6.9e-05 |
| 37 | rs2228083 5.6e-05 | rs12210951 4.2e-05 | rs8000715 5.4e-05 | rs11746937 1.0e-04 | rs765466 5.6e-05 | rs1791652 6.8e-05 | rs520605 4.8e-05 | rs12782902 9.1e-05 | rs633862 9.8e-06 | rs4325188 6.2e-05 | rs920809 6.3e-05 | rs4781357 1.0e-04 | rs3779658 4.3e-05 | rs7533806 8.2e-05 | rs4388294 5.7e-05 | rs9657054 4.0e-05 | rs16951782 7.7e-05 | rs3851596 3.4e-05 | rs7835137 6.7e-05 | rs552581 9.0e-05 | rs17149018 1.8e-05 | rs7179134 7.1e-05 | rs892940 7.4e-05 |
| 38 | rs590974 6.0e-05 | rs2985500 4.4e-05 | rs17059858 5.4e-05 | rs1966625 1.0e-04 | rs9405541 5.8e-05 | rs12186854 7.1e-05 | rs7604061 5.0e-05 | rs698459 9.1e-05 | rs1018109 1.0e-05 | rs11237982 6.3e-05 | rs34365422 6.3e-05 | rs17039448 1.0e-04 | rs7834518 4.6e-05 | rs12376441 8.5e-05 | rs6893444 5.7e-05 | rs10515411 4.0e-05 | rs10497910 7.7e-05 | rs2122113 3.5e-05 | rs11801229 7.0e-05 | rs3911391 9.5e-05 | rs2755140 2.6e-05 | rs11577 7.2e-05 | rs793798 7.4e-05 |
| 39 | rs2938258 6.0e-05 | rs1910653 4.6e-05 | rs2391535 5.6e-05 | rs8038746 1.0e-04 | rs4558315 5.9e-05 | rs2107124 7.2e-05 | rs13098181 5.3e-05 | rs16922827 9.2e-05 | rs12529334 1.4e-05 | rs12582294 6.5e-05 | rs2505903 6.5e-05 | rs2603088 1.0e-04 | rs2150571 4.6e-05 | rs366115 8.6e-05 | rs10733241 6.0e-05 | rs10458871 4.9e-05 | rs10201401 7.8e-05 | rs138053 3.7e-05 | rs6885887 7.1e-05 | rs665743 9.5e-05 | rs131833 3.2e-05 | rs235799 7.6e-05 | rs7856074 7.7e-05 |
| 40 | rs17217105 6.2e-05 | rs1041915 5.1e-05 | rs1036452 5.7e-05 | rs4782493 1.0e-04 | rs12953270 6.0e-05 | rs1486331 7.3e-05 | rs2372972 5.4e-05 | rs3810331 9.3e-05 | rs278364 1.5e-05 | rs4795725 6.7e-05 | rs1990943 6.7e-05 | rs12957915 1.1e-04 | rs2562122 4.9e-05 | rs2486118 8.7e-05 | rs2223930 6.1e-05 | rs9472173 4.9e-05 | rs10516034 8.6e-05 | rs17771505 4.2e-05 | rs6898116 7.2e-05 | rs428826 9.6e-05 | rs10498955 3.3e-05 | rs6903113 8.0e-05 | rs16917364 8.2e-05 |
| 41 | rs11918634 6.2e-05 | rs11594406 5.1e-05 | rs2045566 6.0e-05 | rs2008521 1.1e-04 | rs11169752 6.0e-05 | rs564266 7.4e-05 | rs2603375 5.4e-05 | rs1619656 9.5e-05 | rs3809304 1.6e-05 | rs16902104 6.7e-05 | rs1958351 6.9e-05 | rs10812662 1.1e-04 | rs3751204 4.9e-05 | rs8084409 8.8e-05 | rs7710527 6.2e-05 | rs6658845 5.2e-05 | rs13066227 9.3e-05 | rs10851410 4.3e-05 | rs1893662 7.3e-05 | rs7349861 9.9e-05 | rs1431651 3.7e-05 | rs4527518 8.3e-05 | rs2407122 9.0e-05 |
| 42 | rs2544037 6.4e-05 | rs2064949 5.1e-05 | rs9533567 7.3e-05 | rs2812297 1.1e-04 | rs6016883 6.1e-05 | rs2339703 7.6e-05 | rs2635304 5.7e-05 | rs10150152 9.7e-05 | rs6945781 1.6e-05 | rs7863627 6.8e-05 | rs1209063 7.1e-05 | rs4895290 1.1e-04 | rs2044613 5.2e-05 | rs1496585 9.1e-05 | rs713702 6.3e-05 | rs2038144 5.2e-05 | rs6451310 9.3e-05 | rs7302505 4.9e-05 | rs2648876 7.4e-05 | rs1350085 9.9e-05 | rs6453482 3.8e-05 | rs11615015 8.4e-05 | rs10821792 9.4e-05 |
| 43 | rs11932673 6.4e-05 | rs11238008 5.3e-05 | rs7111879 7.3e-05 | rs4961076 1.1e-04 | rs10409533 6.4e-05 | rs1732293 7.9e-05 | rs2908203 5.8e-05 | rs17773867 1.0e-04 | rs17863795 1.8e-05 | rs4244436 7.0e-05 | rs6427922 7.1e-05 | rs12617277 1.1e-04 | rs8053462 5.4e-05 | rs2867447 9.2e-05 | rs6736318 6.7e-05 | rs7331625 5.3e-05 | rs260019 9.9e-05 | rs2498905 5.0e-05 | rs7600259 7.7e-05 | rs11145626 1.0e-04 | rs2862404 3.8e-05 | rs7350025 8.4e-05 | rs3775580 9.4e-05 |
| 44 | rs246344 6.5e-05 | rs10513066 5.3e-05 | rs2064033 7.8e-05 | rs1542190 1.1e-04 | rs38859 6.4e-05 | rs870585 8.2e-05 | rs4391081 5.8e-05 | rs2075386 1.0e-04 | rs7011172 2.1e-05 | rs2657191 7.1e-05 | rs2075386 7.2e-05 | rs408005 1.1e-04 | rs6082952 5.4e-05 | rs6566587 9.3e-05 | rs3752798 6.8e-05 | rs2267106 5.5e-05 | rs4385147 1.0e-04 | rs4569502 5.1e-05 | rs17580697 8.7e-05 | rs7157717 1.0e-04 | rs9359682 3.9e-05 | rs12633843 8.7e-05 | rs12707089 9.6e-05 |
| 45 | rs13120062 6.6e-05 | rs1752584 5.3e-05 | rs1443438 7.9e-05 | rs1217780 1.1e-04 | rs3912148 6.6e-05 | rs10184453 8.2e-05 | rs6585563 5.9e-05 | rs17651829 1.0e-04 | rs12425617 2.3e-05 | rs333950 7.2e-05 | rs11090262 7.3e-05 | rs11007129 1.2e-04 | rs1507290 5.6e-05 | rs182552 9.3e-05 | rs9328844 6.8e-05 | rs7554044 5.6e-05 | rs12420399 1.0e-04 | rs2071407 5.3e-05 | rs7663838 8.7e-05 | rs13101364 1.1e-04 | rs16876172 4.0e-05 | rs13281070 9.2e-05 | rs3842949 9.8e-05 |
| 46 | rs3741701 6.7e-05 | rs632986 5.5e-05 | rs28756262 8.0e-05 | rs9285466 1.2e-04 | rs10760848 6.7e-05 | rs1838899 8.3e-05 | rs12758184 5.9e-05 | rs9880529 1.1e-04 | rs12742611 2.4e-05 | rs11140511 7.5e-05 | rs1010504 7.4e-05 | rs7289738 1.2e-04 | rs6075982 5.7e-05 | rs4843783 9.4e-05 | rs820363 7.0e-05 | rs7533134 5.6e-05 | rs8059614 1.0e-04 | rs10773692 5.4e-05 | rs16948903 8.9e-05 | rs6472155 1.1e-04 | rs4575879 4.0e-05 | rs7813319 9.2e-05 | rs11609133 9.8e-05 |
| 47 | rs41736 7.3e-05 | rs17299960 5.6e-05 | rs9536041 8.0e-05 | rs12570660 1.2e-04 | rs2153717 7.4e-05 | rs1993392 8.6e-05 | rs7675657 6.1e-05 | rs12629572 1.1e-04 | rs12714151 2.9e-05 | rs10245778 7.5e-05 | rs3861854 7.4e-05 | rs12101488 1.2e-04 | rs2281985 5.7e-05 | rs6504909 9.9e-05 | rs10854884 7.5e-05 | rs12874422 5.7e-05 | rs760794 1.1e-04 | rs9574460 5.4e-05 | rs4593503 9.3e-05 | rs7295752 1.1e-04 | rs9305687 4.1e-05 | rs4668039 9.3e-05 | rs12630450 9.8e-05 |
| 48 | rs12595754 7.4e-05 | rs4545073 5.7e-05 | rs9536049 8.0e-05 | rs9595635 1.2e-04 | rs1204757 7.6e-05 | rs1732304 8.6e-05 | rs4373430 6.2e-05 | rs1041309 1.1e-04 | rs721348 3.1e-05 | rs4395807 7.7e-05 | rs13295392 7.5e-05 | rs150822 1.2e-04 | rs10952779 5.8e-05 | rs4643025 1.0e-04 | rs862943 7.5e-05 | rs10506555 5.7e-05 | rs2882248 1.1e-04 | rs17012581 5.6e-05 | rs4653312 9.4e-05 | rs1178178 1.1e-04 | rs1577237 4.3e-05 | rs7315316 9.6e-05 | rs7999445 1.0e-04 |
| 49 | rs4579577 7.5e-05 | rs132514 5.8e-05 | rs7126331 8.0e-05 | rs745300 1.2e-04 | rs10743555 7.7e-05 | rs1732283 8.9e-05 | rs9814647 6.3e-05 | rs1401063 1.1e-04 | rs6841843 3.4e-05 | rs2654210 7.8e-05 | rs13374557 7.5e-05 | rs1379044 1.3e-04 | rs12371047 6.0e-05 | rs589448 1.0e-04 | rs16934171 7.7e-05 | rs7038767 5.7e-05 | rs172318 1.1e-04 | rs4853241 5.6e-05 | rs2726128 9.6e-05 | rs1206131 1.1e-04 | rs1952896 4.3e-05 | rs2299894 9.7e-05 | rs10766561 1.1e-04 |
| 50 | rs171010 7.6e-05 | rs4958231 5.9e-05 | rs9944229 8.0e-05 | rs12914251 1.3e-04 | rs246344 7.8e-05 | rs469568 8.9e-05 | rs2304881 6.3e-05 | rs4387746 1.1e-04 | rs4519820 3.9e-05 | rs235517 7.9e-05 | rs6585903 7.5e-05 | rs7973974 1.3e-04 | rs6082950 6.1e-05 | rs2250807 1.1e-04 | rs10827211 7.7e-05 | rs4425335 5.9e-05 | rs4701864 1.1e-04 | rs7962254 5.7e-05 | rs4723009 1.0e-04 | rs3812180 1.2e-04 | rs12441564 4.5e-05 | rs235622 9.8e-05 | rs738417 1.1e-04 |
| 51 | rs34365422 8.1e-05 | rs4846479 6.0e-05 | rs3775068 8.1e-05 | rs7702336 1.3e-04 | rs17217105 7.9e-05 | rs321202 9.0e-05 | rs993775 6.4e-05 | rs11980671 1.1e-04 | rs6547632 4.0e-05 | rs7542845 7.9e-05 | rs3865186 8.2e-05 | rs2108258 1.3e-04 | rs12216311 6.2e-05 | rs12962844 1.1e-04 | rs12303320 7.9e-05 | rs4888146 6.4e-05 | rs7933548 1.1e-04 | rs12324717 5.8e-05 | rs7636311 1.0e-04 | rs3812179 1.2e-04 | rs10256677 4.6e-05 | rs11644588 1.0e-04 | rs4972542 1.1e-04 |
| 52 | rs7930308 8.2e-05 | rs17772064 6.2e-05 | rs10807170 8.2e-05 | rs2883645 1.3e-04 | rs1862077 7.9e-05 | rs12928460 9.1e-05 | rs13142826 7.0e-05 | rs10846926 1.2e-04 | rs17868299 4.2e-05 | rs10926354 8.2e-05 | rs10223883 8.7e-05 | rs2199831 1.4e-04 | rs909055 6.3e-05 | rs2676245 1.2e-04 | rs11074939 8.0e-05 | rs7540211 6.4e-05 | rs6697593 1.1e-04 | rs1989696 5.8e-05 | rs9989245 1.0e-04 | rs13184971 1.2e-04 | rs2289754 4.6e-05 | rs1233887 1.0e-04 | rs1417023 1.1e-04 |
| 53 | rs7635110 8.3e-05 | rs132500 6.3e-05 | rs6825946 8.2e-05 | rs9891293 1.3e-04 | rs1421577 8.0e-05 | rs17684388 9.3e-05 | rs322172 7.1e-05 | rs6479222 1.2e-04 | rs11892031 4.3e-05 | rs6826952 8.7e-05 | rs2505171 8.7e-05 | rs1556161 1.4e-04 | rs12186177 6.6e-05 | rs6945041 1.2e-04 | rs7311949 8.3e-05 | rs9529629 6.5e-05 | rs3908834 1.1e-04 | rs7148107 5.9e-05 | rs533259 1.0e-04 | rs2189461 1.3e-04 | rs6575129 4.6e-05 | rs1581995 1.0e-04 | rs3821383 1.1e-04 |
| 54 | rs9391943 8.7e-05 | rs132513 6.3e-05 | rs2968999 8.2e-05 | rs2291219 1.4e-04 | rs7766548 8.1e-05 | rs12519784 9.3e-05 | rs10510908 7.2e-05 | rs16992922 1.2e-04 | rs6990605 4.4e-05 | rs28920 8.9e-05 | rs390201 8.7e-05 | rs1400671 1.5e-04 | rs17106655 7.0e-05 | rs1760058 1.2e-04 | rs10744653 8.8e-05 | rs806366 6.6e-05 | rs17508435 1.2e-04 | rs17012588 6.1e-05 | rs202855 1.0e-04 | rs7242671 1.3e-04 | rs13295392 4.7e-05 | rs11794773 1.1e-04 | rs4799159 1.1e-04 |
| 55 | rs17777278 8.8e-05 | rs1473526 6.4e-05 | rs1436724 8.2e-05 | rs6879032 1.4e-04 | rs7729087 8.4e-05 | rs9845678 9.3e-05 | rs4970972 8.3e-05 | rs207465 1.2e-04 | rs17864670 4.5e-05 | rs1954066 9.1e-05 | rs10139869 9.1e-05 | rs12758064 1.5e-04 | rs853022 7.8e-05 | rs2908201 1.2e-04 | rs12491294 8.9e-05 | rs758250 6.7e-05 | rs4641145 1.2e-04 | rs1335293 6.1e-05 | rs232557 1.0e-04 | rs3812178 1.3e-04 | rs10017065 5.0e-05 | rs6590684 1.1e-04 | rs4905989 1.1e-04 |
| 56 | rs7201995 8.8e-05 | rs11754231 6.8e-05 | rs965513 8.4e-05 | rs10409533 1.4e-04 | rs2836660 8.4e-05 | rs4799856 9.8e-05 | rs8066066 8.3e-05 | rs12148543 1.2e-04 | rs4132608 4.7e-05 | rs12187606 9.3e-05 | rs17111972 9.4e-05 | rs11154583 1.5e-04 | rs3742245 7.9e-05 | rs7021972 1.3e-04 | rs7735749 9.0e-05 | rs12122962 6.9e-05 | rs8082947 1.3e-04 | rs1758726 6.8e-05 | rs1243062 1.0e-04 | rs7696414 1.3e-04 | rs9359686 5.1e-05 | rs2829385 1.1e-04 | rs9822885 1.2e-04 |
| 57 | rs4876200 8.9e-05 | rs480211 6.8e-05 | rs9484314 8.8e-05 | rs17069824 1.4e-04 | rs2250807 8.5e-05 | rs1893409 1.1e-04 | rs11070671 9.1e-05 | rs375154 1.3e-04 | rs481565 4.9e-05 | rs1826956 9.4e-05 | rs1911983 9.4e-05 | rs11636972 1.5e-04 | rs783024 7.9e-05 | rs3913840 1.3e-04 | rs10275188 9.0e-05 | rs2132870 6.9e-05 | rs2160256 1.3e-04 | rs331706 7.0e-05 | rs7521055 1.0e-04 | rs17512574 1.3e-04 | rs2490215 5.7e-05 | rs1766967 1.1e-04 | rs4112315 1.2e-04 |
| 58 | rs1055492 9.3e-05 | rs9347799 6.9e-05 | rs1356370 9.0e-05 | rs7647575 1.5e-04 | rs12810700 8.7e-05 | rs212087 1.1e-04 | rs6061366 9.5e-05 | rs11847083 1.3e-04 | rs8079626 5.0e-05 | rs2655217 9.6e-05 | rs10920260 9.6e-05 | rs10770062 1.6e-04 | rs701565 8.4e-05 | rs10001415 1.4e-04 | rs6501554 9.2e-05 | rs7784844 7.0e-05 | rs2024629 1.3e-04 | rs1060291 7.4e-05 | rs485325 1.1e-04 | rs2215170 1.3e-04 | rs6699043 6.1e-05 | rs362842 1.1e-04 | rs7104791 1.2e-04 |
| 59 | rs16967692 9.3e-05 | rs17817803 7.0e-05 | rs11154288 9.2e-05 | rs1499676 1.5e-04 | rs930295 8.7e-05 | rs11085420 1.1e-04 | rs7830124 9.7e-05 | rs899541 1.3e-04 | rs4270726 5.2e-05 | rs6565620 9.8e-05 | rs2962896 9.7e-05 | rs717139 1.6e-04 | rs4660036 8.5e-05 | rs13116766 1.4e-04 | rs1421577 9.5e-05 | rs12206944 7.4e-05 | rs6785948 1.4e-04 | rs1422268 7.4e-05 | rs12459996 1.1e-04 | rs961777 1.4e-04 | rs10888249 6.1e-05 | rs165598 1.1e-04 | rs974788 1.2e-04 |
| 60 | rs7307294 9.5e-05 | rs5750695 7.0e-05 | rs3863288 9.6e-05 | rs2028889 1.5e-04 | rs11871984 8.8e-05 | rs1445523 1.1e-04 | rs4407889 1.0e-04 | rs929878 1.4e-04 | rs7606254 5.2e-05 | rs6547632 9.9e-05 | rs349353 1.0e-04 | rs11037611 1.6e-04 | rs465663 8.5e-05 | rs11720066 1.4e-04 | rs7906793 9.7e-05 | rs1278384 7.7e-05 | rs2223361 1.4e-04 | rs4946265 7.9e-05 | rs2486669 1.1e-04 | rs9320410 1.4e-04 | rs8116456 6.2e-05 | rs2242637 1.1e-04 | rs1945119 1.2e-04 |
| 61 | rs6433738 9.7e-05 | rs9533044 7.1e-05 | rs2098747 9.7e-05 | rs1968441 1.5e-04 | rs9558485 9.0e-05 | rs1345663 1.1e-04 | rs1413106 1.0e-04 | rs10991043 1.4e-04 | rs552148 5.4e-05 | rs2051649 1.0e-04 | rs2268433 1.1e-04 | rs1123203 1.6e-04 | rs13248954 8.5e-05 | rs11070671 1.5e-04 | rs6459484 9.7e-05 | rs12475034 7.8e-05 | rs7573313 1.4e-04 | rs161041 8.1e-05 | rs8081534 1.1e-04 | rs2117896 1.4e-04 | rs4511370 6.3e-05 | rs1548440 1.1e-04 | rs1693892 1.2e-04 |
| 62 | rs6717927 9.9e-05 | rs7522641 7.2e-05 | rs4148654 9.7e-05 | rs2434349 1.5e-04 | rs17229238 9.1e-05 | rs3743205 1.1e-04 | rs11928474 1.0e-04 | rs2568609 1.4e-04 | rs1404143 5.5e-05 | rs2654673 1.0e-04 | rs624285 1.1e-04 | rs2075386 1.6e-04 | rs826460 8.5e-05 | rs16961885 1.5e-04 | rs1056897 9.7e-05 | rs9405157 7.8e-05 | rs10945881 1.5e-04 | rs2039553 8.1e-05 | rs6817250 1.1e-04 | rs2399955 1.4e-04 | rs13157486 6.4e-05 | rs2267846 1.1e-04 | rs4756999 1.2e-04 |
| 63 | rs1862077 1.0e-04 | rs10913713 7.2e-05 | rs10759944 9.8e-05 | rs1982942 1.6e-04 | rs10509830 9.2e-05 | rs2585193 1.1e-04 | rs9919652 1.1e-04 | rs2834763 1.4e-04 | rs2224437 5.5e-05 | rs1367324 1.0e-04 | rs6465973 1.1e-04 | rs10513066 1.6e-04 | rs9849779 8.5e-05 | rs775876 1.5e-04 | rs16901546 9.7e-05 | rs7089520 8.0e-05 | rs978875 1.5e-04 | rs7006781 8.4e-05 | rs12520537 1.1e-04 | rs1553499 1.4e-04 | rs9529475 6.4e-05 | rs17647464 1.1e-04 | rs8006648 1.2e-04 |
| 64 | rs4383453 1.0e-04 | rs1948853 7.5e-05 | rs7983232 9.8e-05 | rs12966991 1.6e-04 | rs381726 9.4e-05 | rs2661406 1.2e-04 | rs534654 1.1e-04 | rs9544872 1.5e-04 | rs4301169 5.5e-05 | rs7762994 1.0e-04 | rs195853 1.1e-04 | rs17539401 1.6e-04 | rs17124965 8.7e-05 | rs2618108 1.5e-04 | rs2863231 9.8e-05 | rs7237245 8.1e-05 | rs4772116 1.5e-04 | rs11005008 8.6e-05 | rs4427776 1.1e-04 | rs12431517 1.5e-04 | rs7781788 6.5e-05 | rs8096392 1.1e-04 | rs159091 1.2e-04 |
| 65 | rs3930083 1.0e-04 | rs8046350 8.0e-05 | rs4875832 1.0e-04 | rs10809012 1.6e-04 | rs1574182 9.6e-05 | rs10845690 1.2e-04 | rs746886 1.1e-04 | rs2550766 1.5e-04 | rs9907106 5.6e-05 | rs267527 1.1e-04 | rs3738290 1.1e-04 | rs17539942 1.6e-04 | rs9854404 9.1e-05 | rs6076466 1.5e-04 | rs2421932 9.8e-05 | rs1870327 9.1e-05 | rs6911763 1.6e-04 | rs2802275 8.8e-05 | rs11142623 1.2e-04 | rs963402 1.5e-04 | rs181559 6.6e-05 | rs12507919 1.1e-04 | rs6979491 1.2e-04 |
| 66 | rs38859 1.1e-04 | rs2298948 8.1e-05 | rs1459377 1.0e-04 | rs643301 1.6e-04 | rs10954144 9.6e-05 | rs2875859 1.2e-04 | rs17043133 1.1e-04 | rs17824897 1.5e-04 | rs2284021 5.6e-05 | rs920809 1.1e-04 | rs10847284 1.1e-04 | rs16942055 1.7e-04 | rs9858014 9.1e-05 | rs2066362 1.5e-04 | rs6599685 9.9e-05 | rs6051639 9.1e-05 | rs17794487 1.6e-04 | rs10056714 8.9e-05 | rs6536958 1.2e-04 | rs7110565 1.5e-04 | rs1881417 6.8e-05 | rs10493770 1.2e-04 | rs10756653 1.3e-04 |
| 67 | rs2153717 1.1e-04 | rs6056973 8.1e-05 | rs1364301 1.0e-04 | rs7632091 1.7e-04 | rs4388294 9.8e-05 | rs1860218 1.2e-04 | rs748731 1.1e-04 | rs11681748 1.6e-04 | rs2292001 5.8e-05 | rs6493397 1.1e-04 | rs11982522 1.1e-04 | rs208807 1.7e-04 | rs11693950 9.1e-05 | rs7216474 1.5e-04 | rs2834932 1.0e-04 | rs6599685 9.2e-05 | rs12427618 1.6e-04 | rs13422220 9.0e-05 | rs3823720 1.2e-04 | rs701716 1.5e-04 | rs227030 6.9e-05 | rs2190208 1.2e-04 | rs4737274 1.3e-04 |
| 68 | rs12292704 1.1e-04 | rs4735683 8.6e-05 | rs7319451 1.0e-04 | rs6576373 1.7e-04 | rs6517254 1.0e-04 | rs4770399 1.2e-04 | rs38892 1.1e-04 | rs6955705 1.6e-04 | rs2589223 5.8e-05 | rs7743815 1.1e-04 | rs861070 1.1e-04 | rs11746937 1.7e-04 | rs7332893 9.3e-05 | rs7017975 1.5e-04 | rs38859 1.0e-04 | rs4589285 9.2e-05 | rs742115 1.7e-04 | rs1934225 9.1e-05 | rs12155213 1.2e-04 | rs884844 1.5e-04 | rs1629521 7.1e-05 | rs17202902 1.2e-04 | rs12953828 1.3e-04 |
| 69 | rs6017227 1.1e-04 | rs6918826 9.2e-05 | rs7850258 1.0e-04 | rs8004267 1.7e-04 | rs7732107 1.0e-04 | rs7213894 1.3e-04 | rs11251694 1.1e-04 | rs4682144 1.6e-04 | rs6987719 5.8e-05 | rs17751492 1.1e-04 | rs10103353 1.1e-04 | rs17456745 1.7e-04 | rs2131495 9.4e-05 | rs1195261 1.6e-04 | rs743168 1.0e-04 | rs7747591 9.5e-05 | rs4133297 1.7e-04 | rs2899051 9.1e-05 | rs13002075 1.3e-04 | rs6442700 1.5e-04 | rs240664 7.1e-05 | rs17300166 1.2e-04 | rs965235 1.3e-04 |
| 70 | rs3743807 1.2e-04 | rs12489095 9.3e-05 | rs9536184 1.1e-04 | rs2239961 1.7e-04 | rs12292704 1.0e-04 | rs4926674 1.3e-04 | rs17023493 1.1e-04 | rs3806708 1.6e-04 | rs2576997 5.9e-05 | rs6679827 1.1e-04 | rs13181346 1.1e-04 | rs8094095 1.8e-04 | rs6984900 9.7e-05 | rs2729682 1.7e-04 | rs2269096 1.0e-04 | rs9541994 9.8e-05 | rs11707556 1.8e-04 | rs10454039 9.1e-05 | rs4962656 1.4e-04 | rs12359499 1.5e-04 | rs11025356 7.5e-05 | rs2057916 1.2e-04 | rs11851301 1.4e-04 |
| 71 | rs2292867 1.2e-04 | rs7534162 9.4e-05 | rs2699376 1.1e-04 | rs2968005 1.8e-04 | rs1996291 1.0e-04 | rs1774037 1.3e-04 | rs1463851 1.2e-04 | rs7543281 1.6e-04 | rs1866093 5.9e-05 | rs392715 1.2e-04 | rs10920269 1.2e-04 | rs10882146 1.8e-04 | rs1536337 1.0e-04 | rs4408298 1.7e-04 | rs930295 1.0e-04 | rs9458548 9.9e-05 | rs754689 1.8e-04 | rs8073426 9.2e-05 | rs10263645 1.4e-04 | rs7932763 1.5e-04 | rs1881336 7.5e-05 | rs7749161 1.2e-04 | rs10810355 1.4e-04 |
| 72 | rs713702 1.2e-04 | rs2295135 9.6e-05 | rs6972287 1.1e-04 | rs8025033 1.9e-04 | rs11709020 1.0e-04 | rs329317 1.3e-04 | rs16862250 1.2e-04 | rs12407970 1.7e-04 | rs1473591 6.1e-05 | rs6581328 1.2e-04 | rs6585910 1.2e-04 | rs7618885 1.8e-04 | rs10893243 1.0e-04 | rs11132255 1.8e-04 | rs17010981 1.0e-04 | rs9328844 1.0e-04 | rs11193663 1.8e-04 | rs6426280 9.5e-05 | rs12253675 1.4e-04 | rs12279811 1.5e-04 | rs331147 7.6e-05 | rs17627345 1.2e-04 | rs837329 1.4e-04 |
| 73 | rs17096085 1.2e-04 | rs13438451 9.7e-05 | rs3927511 1.1e-04 | rs855508 1.9e-04 | rs6103533 1.0e-04 | rs2052329 1.3e-04 | rs10225959 1.2e-04 | rs729986 1.7e-04 | rs2440684 6.1e-05 | rs17824897 1.2e-04 | rs8063248 1.2e-04 | rs7107803 1.9e-04 | rs9817455 1.0e-04 | rs11532670 1.8e-04 | rs1914695 1.0e-04 | rs7906793 1.0e-04 | rs4660036 1.8e-04 | rs633862 9.6e-05 | rs7489477 1.4e-04 | rs4466848 1.6e-04 | rs10904728 7.7e-05 | rs849315 1.2e-04 | rs968778 1.5e-04 |
| 74 | rs2250807 1.2e-04 | rs7302629 9.7e-05 | rs12019764 1.1e-04 | rs11756584 1.9e-04 | rs6016884 1.1e-04 | rs7088764 1.3e-04 | rs366115 1.2e-04 | rs6914034 1.8e-04 | rs887844 6.2e-05 | rs9688645 1.2e-04 | rs325260 1.2e-04 | rs6551250 1.9e-04 | rs12416632 1.1e-04 | rs6845801 1.8e-04 | rs4238357 1.1e-04 | rs2804665 1.0e-04 | rs7190101 1.9e-04 | rs7901534 9.7e-05 | rs9304434 1.4e-04 | rs10929808 1.6e-04 | rs1374818 8.0e-05 | rs1610041 1.2e-04 | rs800729 1.5e-04 |
| 75 | rs603852 1.3e-04 | rs10746348 9.7e-05 | rs1421602 1.1e-04 | rs667168 1.9e-04 | rs1399221 1.1e-04 | rs4609193 1.4e-04 | rs10513642 1.3e-04 | rs1411101 1.8e-04 | rs17862859 6.3e-05 | rs2184747 1.2e-04 | rs1294506 1.2e-04 | rs1051288 1.9e-04 | rs1641674 1.1e-04 | rs12229047 1.8e-04 | rs7715602 1.1e-04 | rs244660 1.0e-04 | rs7023309 1.9e-04 | rs1147857 9.7e-05 | rs10032551 1.4e-04 | rs12999289 1.6e-04 | rs16981520 8.3e-05 | rs1941213 1.2e-04 | rs4964109 1.5e-04 |
| 76 | rs11742884 1.3e-04 | rs11116966 9.8e-05 | rs9535894 1.1e-04 | rs12359035 1.9e-04 | rs318497 1.1e-04 | rs2359797 1.4e-04 | rs11916217 1.3e-04 | rs952004 1.8e-04 | rs17868338 6.9e-05 | rs1970533 1.2e-04 | rs17636626 1.2e-04 | rs2979750 2.0e-04 | rs10893249 1.1e-04 | rs3792608 1.8e-04 | rs9614363 1.1e-04 | rs8099603 1.0e-04 | rs7072670 1.9e-04 | rs9963409 9.9e-05 | rs10978504 1.4e-04 | rs773358 1.6e-04 | rs2627525 8.3e-05 | rs1995830 1.2e-04 | rs9868179 1.5e-04 |
| 77 | rs28444486 1.3e-04 | rs9283872 1.0e-04 | rs610902 1.2e-04 | rs899541 2.0e-04 | rs10092374 1.1e-04 | rs1542190 1.4e-04 | rs1413107 1.3e-04 | rs1490070 1.9e-04 | rs7957137 7.0e-05 | rs4847034 1.3e-04 | rs2161416 1.3e-04 | rs13380014 2.0e-04 | rs4779829 1.1e-04 | rs2908203 1.8e-04 | rs2250807 1.1e-04 | rs11593943 1.0e-04 | rs12516704 1.9e-04 | rs4853700 1.0e-04 | rs6734275 1.4e-04 | rs7622713 1.6e-04 | rs12550268 8.6e-05 | rs4337384 1.3e-04 | rs645718 1.5e-04 |
| 78 | rs2836660 1.4e-04 | rs820363 1.0e-04 | rs10770871 1.2e-04 | rs4804978 2.0e-04 | rs1807445 1.2e-04 | rs1998067 1.4e-04 | rs182552 1.3e-04 | rs1281603 1.9e-04 | rs17247805 7.1e-05 | rs4740884 1.3e-04 | rs11838607 1.3e-04 | rs10804418 2.0e-04 | rs2518216 1.2e-04 | rs1916286 1.8e-04 | rs2368053 1.2e-04 | rs1521753 1.1e-04 | rs12897699 2.0e-04 | rs10875223 1.0e-04 | rs6998540 1.5e-04 | rs11872163 1.7e-04 | rs17677071 8.7e-05 | rs2604251 1.4e-04 | rs10053266 1.5e-04 |
| 79 | rs7914615 1.4e-04 | rs2295527 1.1e-04 | rs1284879 1.2e-04 | rs1838899 2.0e-04 | rs7307294 1.2e-04 | rs1076235 1.5e-04 | rs282413 1.3e-04 | rs1209063 1.9e-04 | rs9368002 7.9e-05 | rs2425502 1.3e-04 | rs12231740 1.4e-04 | rs6879032 2.0e-04 | rs987811 1.2e-04 | rs7314262 1.8e-04 | rs7424428 1.2e-04 | rs8070464 1.1e-04 | rs7692931 2.0e-04 | rs2095470 1.0e-04 | rs7166598 1.5e-04 | rs4724613 1.7e-04 | rs10095860 8.9e-05 | rs9845309 1.4e-04 | rs6538703 1.5e-04 |
| 80 | rs7934757 1.4e-04 | rs7402977 1.1e-04 | rs10770872 1.2e-04 | rs6538538 2.1e-04 | rs7546764 1.2e-04 | rs1321704 1.5e-04 | rs4605456 1.3e-04 | rs2604376 1.9e-04 | rs6710665 8.2e-05 | rs4881232 1.3e-04 | rs10176047 1.4e-04 | rs6016884 2.0e-04 | rs2760311 1.2e-04 | rs393115 1.8e-04 | rs10999846 1.2e-04 | rs260718 1.1e-04 | rs4953085 2.0e-04 | rs7961510 1.1e-04 | rs12179836 1.5e-04 | rs10954734 1.7e-04 | rs1123923 8.9e-05 | rs2284210 1.4e-04 | rs3848361 1.6e-04 |
| 81 | rs7520333 1.4e-04 | rs12322464 1.1e-04 | rs17834626 1.2e-04 | rs1209063 2.2e-04 | rs6030450 1.2e-04 | rs918377 1.5e-04 | rs721316 1.3e-04 | rs7956979 1.9e-04 | rs4629472 8.2e-05 | rs17622419 1.4e-04 | rs8079626 1.4e-04 | rs1890629 2.0e-04 | rs955667 1.2e-04 | rs10183087 1.8e-04 | rs4982973 1.2e-04 | rs260735 1.1e-04 | rs11033618 2.0e-04 | rs4897080 1.1e-04 | rs10408886 1.5e-04 | rs973214 1.7e-04 | rs9342631 9.0e-05 | rs1043235 1.4e-04 | rs300559 1.6e-04 |
| 82 | rs17295401 1.4e-04 | rs2547958 1.1e-04 | rs10492790 1.2e-04 | rs2131495 2.2e-04 | rs2537978 1.2e-04 | rs335644 1.5e-04 | rs2282624 1.4e-04 | rs35974282 1.9e-04 | rs1528873 8.3e-05 | rs3750213 1.4e-04 | rs10070091 1.4e-04 | rs714789 2.1e-04 | rs984664 1.2e-04 | rs6581889 1.8e-04 | rs2568453 1.3e-04 | rs208807 1.1e-04 | rs8093884 2.0e-04 | rs331726 1.1e-04 | rs981272 1.6e-04 | rs4243847 1.7e-04 | rs1958351 9.1e-05 | rs6053046 1.4e-04 | rs1837700 1.6e-04 |
| 83 | rs4889107 1.4e-04 | rs3912317 1.1e-04 | rs3099468 1.3e-04 | rs2000316 2.2e-04 | rs12758064 1.2e-04 | rs1979450 1.5e-04 | rs2218433 1.4e-04 | rs9382621 1.9e-04 | rs9383052 8.4e-05 | rs12129498 1.4e-04 | rs12877121 1.4e-04 | rs11222888 2.1e-04 | rs4333906 1.2e-04 | rs6691351 1.8e-04 | rs2414058 1.3e-04 | rs10976384 1.1e-04 | rs7591705 2.0e-04 | rs3815948 1.1e-04 | rs9899611 1.6e-04 | rs4254372 1.7e-04 | rs7117703 9.1e-05 | rs36094861 1.4e-04 | rs24023 1.6e-04 |
| 84 | rs12926289 1.4e-04 | rs10132666 1.1e-04 | rs7303758 1.3e-04 | rs7712162 2.3e-04 | rs7706863 1.2e-04 | rs323990 1.5e-04 | rs16969661 1.4e-04 | rs2927322 1.9e-04 | rs4676093 8.4e-05 | rs6016958 1.4e-04 | rs11659563 1.4e-04 | rs7720606 2.1e-04 | rs1509939 1.2e-04 | rs9809592 1.9e-04 | rs7635110 1.3e-04 | rs1892143 1.1e-04 | rs2433431 2.1e-04 | rs10115457 1.1e-04 | rs4937030 1.6e-04 | rs12275038 1.7e-04 | rs2193516 9.2e-05 | rs11768279 1.5e-04 | rs16854319 1.6e-04 |
| 85 | rs6564735 1.4e-04 | rs10132731 1.1e-04 | rs7019219 1.3e-04 | rs1515215 2.3e-04 | rs12595754 1.2e-04 | rs235312 1.6e-04 | rs4978629 1.4e-04 | rs726256 1.9e-04 | rs3885983 8.5e-05 | rs9321763 1.5e-04 | rs4364907 1.4e-04 | rs2643998 2.1e-04 | rs1761741 1.2e-04 | rs17022006 1.9e-04 | rs2302248 1.3e-04 | rs244684 1.1e-04 | rs9609757 2.1e-04 | rs10511954 1.2e-04 | rs1292043 1.6e-04 | rs4623911 1.7e-04 | rs7151079 9.2e-05 | rs4320401 1.5e-04 | rs11773055 1.7e-04 |
| 86 | rs3918337 1.5e-04 | rs17095545 1.1e-04 | rs9536067 1.3e-04 | rs1466344 2.4e-04 | rs1317981 1.2e-04 | rs6945041 1.6e-04 | rs6807050 1.5e-04 | rs1866822 1.9e-04 | rs2284022 8.9e-05 | rs1619656 1.5e-04 | rs796038 1.4e-04 | rs1061808 2.1e-04 | rs1325992 1.3e-04 | rs11131228 1.9e-04 | rs10974470 1.3e-04 | rs4898926 1.1e-04 | rs766366 2.1e-04 | rs2353776 1.2e-04 | rs575775 1.6e-04 | rs11078840 1.8e-04 | rs12152542 9.3e-05 | rs178029 1.5e-04 | rs3861665 1.7e-04 |
| 87 | rs9405541 1.5e-04 | rs13325414 1.1e-04 | rs9536077 1.3e-04 | rs6945781 2.4e-04 | rs2235749 1.2e-04 | rs10271373 1.6e-04 | rs9996938 1.5e-04 | rs17059066 2.0e-04 | rs2963155 9.0e-05 | rs12714151 1.5e-04 | rs2290037 1.5e-04 | rs17650296 2.1e-04 | rs11988623 1.3e-04 | rs4404254 1.9e-04 | rs7247504 1.3e-04 | rs7149011 1.2e-04 | rs1336850 2.1e-04 | rs732289 1.2e-04 | rs7048339 1.7e-04 | rs9304382 1.8e-04 | rs7609997 9.4e-05 | rs2829368 1.5e-04 | rs9950784 1.7e-04 |
| 88 | rs6564734 1.5e-04 | rs10139921 1.1e-04 | rs9526957 1.3e-04 | rs10122919 2.4e-04 | rs7201995 1.3e-04 | rs1556591 1.6e-04 | rs10483014 1.5e-04 | rs648384 2.0e-04 | rs1618092 9.1e-05 | rs1972597 1.5e-04 | rs607301 1.5e-04 | rs920035 2.2e-04 | rs11694519 1.3e-04 | rs2153934 1.9e-04 | rs10501068 1.3e-04 | rs13279605 1.2e-04 | rs733970 2.1e-04 | rs10861085 1.2e-04 | rs17787522 1.7e-04 | rs9881237 1.8e-04 | rs1875931 9.6e-05 | rs4963666 1.6e-04 | rs6069073 1.7e-04 |
| 89 | rs1978752 1.5e-04 | rs11860580 1.2e-04 | rs581170 1.3e-04 | rs1871567 2.4e-04 | rs1419424 1.3e-04 | rs3750889 1.6e-04 | rs16969660 1.5e-04 | rs6822760 2.1e-04 | rs2022068 9.3e-05 | rs207465 1.5e-04 | rs432177 1.6e-04 | rs9350354 2.2e-04 | rs1397023 1.3e-04 | rs2704102 1.9e-04 | rs7300543 1.3e-04 | rs2033538 1.2e-04 | rs2893820 2.2e-04 | rs1552336 1.2e-04 | rs4916524 1.7e-04 | rs1178163 1.8e-04 | rs170544 9.7e-05 | rs17601584 1.6e-04 | rs4433946 1.7e-04 |
| 90 | rs542535 1.5e-04 | rs3746429 1.3e-04 | rs11885078 1.3e-04 | rs2209778 2.4e-04 | rs9539866 1.3e-04 | rs17147527 1.6e-04 | rs1418795 1.6e-04 | rs1982942 2.1e-04 | rs17517624 9.4e-05 | rs7153453 1.5e-04 | rs11748886 1.6e-04 | rs6016883 2.3e-04 | rs3752611 1.3e-04 | rs4656381 2.0e-04 | rs10746030 1.3e-04 | rs6517254 1.2e-04 | rs9366648 2.2e-04 | rs12044804 1.2e-04 | rs17623893 1.7e-04 | rs17668453 1.8e-04 | rs7529313 9.7e-05 | rs13152028 1.6e-04 | rs2616133 1.7e-04 |
| 91 | rs2209592 1.5e-04 | rs2295525 1.3e-04 | rs9536061 1.4e-04 | rs2583636 2.4e-04 | rs10836529 1.4e-04 | rs10803773 1.6e-04 | rs12929101 1.6e-04 | rs964401 2.1e-04 | rs11090262 9.6e-05 | rs6889431 1.5e-04 | rs7822849 1.6e-04 | rs1041309 2.3e-04 | rs17421240 1.3e-04 | rs2899593 2.0e-04 | rs7977104 1.4e-04 | rs28641816 1.2e-04 | rs17079031 2.2e-04 | rs9920859 1.2e-04 | rs907956 1.8e-04 | rs2904155 1.9e-04 | rs17818667 9.7e-05 | rs17493657 1.6e-04 | rs6881702 1.7e-04 |
| 92 | rs11580092 1.5e-04 | rs10032294 1.3e-04 | rs9526890 1.4e-04 | rs2681017 2.4e-04 | rs1159741 1.4e-04 | rs17162231 1.7e-04 | rs9976987 1.6e-04 | rs9350354 2.2e-04 | rs1515592 9.7e-05 | rs7537011 1.6e-04 | rs17636389 1.6e-04 | rs8013602 2.3e-04 | rs11666805 1.3e-04 | rs9932893 2.1e-04 | rs12077203 1.4e-04 | rs4888764 1.2e-04 | rs10819626 2.2e-04 | rs11869174 1.3e-04 | rs8178721 1.8e-04 | rs1016188 1.9e-04 | rs220409 1.1e-04 | rs474342 1.7e-04 | rs1369285 1.7e-04 |
| 93 | rs6773564 1.5e-04 | rs30708 1.3e-04 | rs4962447 1.4e-04 | rs7586848 2.4e-04 | rs6496898 1.4e-04 | rs13273810 1.7e-04 | rs4741030 1.6e-04 | rs8094095 2.2e-04 | rs4820254 1.0e-04 | rs4324894 1.6e-04 | rs7513206 1.6e-04 | rs17765985 2.3e-04 | rs2050719 1.4e-04 | rs12758184 2.1e-04 | rs13187359 1.4e-04 | rs1551655 1.2e-04 | rs4916255 2.3e-04 | rs38204 1.3e-04 | rs1983314 1.8e-04 | rs6089669 1.9e-04 | rs2715730 1.1e-04 | rs10212213 1.7e-04 | rs1003599 1.8e-04 |
| 94 | rs7546764 1.6e-04 | rs10743555 1.3e-04 | rs11732323 1.4e-04 | rs9972279 2.4e-04 | rs4812640 1.4e-04 | rs3901667 1.7e-04 | rs721607 1.6e-04 | rs8016998 2.2e-04 | rs9871288 1.0e-04 | rs7137336 1.6e-04 | rs10521067 1.7e-04 | rs7711479 2.3e-04 | rs6907923 1.4e-04 | rs1559931 2.1e-04 | rs9614362 1.4e-04 | rs10808897 1.2e-04 | rs11749542 2.3e-04 | rs10851411 1.3e-04 | rs7662632 1.8e-04 | rs2296937 1.9e-04 | rs1507966 1.1e-04 | rs2266953 1.7e-04 | rs486840 1.8e-04 |
| 95 | rs10743555 1.6e-04 | rs6711052 1.3e-04 | rs10743426 1.4e-04 | rs6551250 2.4e-04 | rs6995874 1.4e-04 | rs9579205 1.8e-04 | rs11735191 1.6e-04 | rs1542190 2.3e-04 | rs10221110 1.0e-04 | rs10904368 1.6e-04 | rs7187154 1.7e-04 | rs12903021 2.3e-04 | rs7307365 1.4e-04 | rs4761154 2.1e-04 | rs6909815 1.5e-04 | rs10815530 1.2e-04 | rs11685771 2.3e-04 | rs4739523 1.3e-04 | rs481236 1.8e-04 | rs2241692 1.9e-04 | rs4405588 1.1e-04 | rs2817318 1.7e-04 | rs858952 1.8e-04 |
| 96 | rs7766548 1.6e-04 | rs10993787 1.4e-04 | rs10841914 1.5e-04 | rs2840354 2.5e-04 | rs1056897 1.4e-04 | rs12942387 1.8e-04 | rs12599236 1.7e-04 | rs9376660 2.3e-04 | rs718400 1.1e-04 | rs375154 1.6e-04 | rs7941534 1.7e-04 | rs12595510 2.3e-04 | rs1192658 1.4e-04 | rs750666 2.1e-04 | rs10208636 1.5e-04 | rs720682 1.2e-04 | rs2747160 2.3e-04 | rs470093 1.3e-04 | rs7902910 1.9e-04 | rs1461898 1.9e-04 | rs6803772 1.1e-04 | rs2829344 1.7e-04 | rs2339461 1.8e-04 |
| 97 | rs11086913 1.6e-04 | rs1006812 1.4e-04 | rs571241 1.5e-04 | rs9595638 2.5e-04 | rs2834939 1.4e-04 | rs4239767 1.8e-04 | rs861588 1.7e-04 | rs866339 2.3e-04 | rs6454284 1.1e-04 | rs537172 1.6e-04 | rs6806286 1.7e-04 | rs7702336 2.3e-04 | rs12622452 1.4e-04 | rs2488062 2.1e-04 | rs41756 1.5e-04 | rs1278396 1.3e-04 | rs11673382 2.3e-04 | rs8063994 1.3e-04 | rs2180768 2.0e-04 | rs16847544 1.9e-04 | rs6986444 1.2e-04 | rs7573476 1.7e-04 | rs6929221 1.8e-04 |
| 98 | rs17362588 1.6e-04 | rs3739642 1.4e-04 | rs465880 1.5e-04 | rs11747598 2.5e-04 | rs2414058 1.4e-04 | rs3804141 1.9e-04 | rs1004622 1.7e-04 | rs4487812 2.3e-04 | rs1783280 1.1e-04 | rs10892923 1.7e-04 | rs4949444 1.7e-04 | rs34365422 2.4e-04 | rs10790691 1.4e-04 | rs2305641 2.1e-04 | rs7763703 1.5e-04 | rs12070417 1.3e-04 | rs7085170 2.4e-04 | rs17633650 1.3e-04 | rs4798085 2.0e-04 | rs1766467 1.9e-04 | rs11066776 1.2e-04 | rs4629769 1.7e-04 | rs745351 1.9e-04 |
| 99 | rs2148709 1.6e-04 | rs1001408 1.4e-04 | rs11640249 1.5e-04 | rs732442 2.5e-04 | rs7765600 1.4e-04 | rs11641366 1.9e-04 | rs840385 1.8e-04 | rs9291123 2.3e-04 | rs4149172 1.1e-04 | rs2546082 1.7e-04 | rs369982 1.8e-04 | rs12053303 2.4e-04 | rs1474664 1.4e-04 | rs9814647 2.1e-04 | rs2447924 1.5e-04 | rs10145625 1.3e-04 | rs7476649 2.4e-04 | rs4669988 1.3e-04 | rs7438808 2.0e-04 | rs12331925 1.9e-04 | rs10872715 1.2e-04 | rs11190831 1.7e-04 | rs1862070 1.9e-04 |
| 100 | rs11169752 1.6e-04 | rs2108258 1.4e-04 | rs2952862 1.5e-04 | rs12455030 2.5e-04 | rs7520333 1.5e-04 | rs12409228 1.9e-04 | rs8126935 1.8e-04 | rs7492368 2.3e-04 | rs6534321 1.1e-04 | rs9861503 1.7e-04 | rs564266 1.8e-04 | rs1563497 2.4e-04 | rs10920260 1.4e-04 | rs12997380 2.2e-04 | rs2834939 1.5e-04 | rs516834 1.3e-04 | rs12945384 2.4e-04 | rs17707070 1.3e-04 | rs9442387 2.1e-04 | rs7610626 2.0e-04 | rs4346059 1.2e-04 | rs2248829 1.8e-04 | rs1421018 1.9e-04 |
